# Anomaly Detection in Time Series Data Using Reversible Instance Normalized Anomaly Transformer

**DOI:** 10.3390/s23229272

**Published:** 2023-11-19

**Authors:** Ranjai Baidya, Heon Jeong

**Affiliations:** 1 Kpro System, 673-1 Dogok-ri, Wabu-eup, Namyangju-si 12270, Gyeonggi-do, Republic of Korea; ranjai123baidya@gmail.com; 2 Department of Fire Service Administration, Chodang University, 80, Muanro, Muaneup, Muangun 58530, Jeollanam-do, Republic of Korea

**Keywords:** time series data, anomaly detection, attention mechanism, transformer, normalization

## Abstract

Anomalies are infrequent in nature, but detecting these anomalies could be crucial for the proper functioning of any system. The rarity of anomalies could be a challenge for their detection as detection models are required to depend on the relations of the datapoints with their adjacent datapoints. In this work, we use the rarity of anomalies to detect them. For this, we introduce the reversible instance normalized anomaly transformer (RINAT). Rooted in the foundational principles of the anomaly transformer, RINAT incorporates both prior and series associations for each time point. The prior association uses a learnable Gaussian kernel to ensure a thorough understanding of the adjacent concentration inductive bias. In contrast, the series association method uses self-attention techniques to specifically focus on the original raw data. Furthermore, because anomalies are rare in nature, we utilize normalized data to identify series associations and employ non-normalized data to uncover prior associations. This approach enhances the modelled series associations and, consequently, improves the association discrepancies.

## 1. Introduction

Anomaly detection in time series data is pivotal in modern data analysis [1] and involves identifying rare patterns or discrepancies that deviate from expected behaviors. This form of detection has a broad range of applications in industries, such as manufacturing, healthcare, and finance [2,3,4]. As technological advancements continue, we are producing and collecting more data than ever before. This influx is not just about the sheer volume of data but also its complexity. Complex relationships and patterns embedded within data grow more nuanced as data expand. Given that many modern systems and processes are data-driven, even minor irregularities can lead to significant consequences [5].

Not all anomalies are of concern; some might be benign outliers without substantial impacts. However, others could indicate severe issues, such as critical system failures. In industries, like system operations, finance, and healthcare, distinguishing between these types of anomalies can be of paramount importance [6,7,8].

We analyze time series data either as univariate or multivariate [9]. These data can be further decomposed into four distinct types [10]. The secular trend represents the consistent, long-term direction of a dataset. Seasonal variations are predictable patterns that recur at regular intervals, like sales spikes during holidays. Cyclical fluctuations refer to longer-term changes without a fixed pattern, often influenced by broader conditions, like economic recessions. Irregular variations represent unpredictable changes due to unforeseen events or outliers, with irregular anomalies being these sudden, unexpected variations.

In this paper, we suggest the reversible instance normalized anomaly transformer for unsupervised anomaly detection in real-life time series data. First, we considered the transformer [11] architecture, following the success of the anomaly transformer [12] in anomaly detection. Transformers have also achieved positive results in the areas of natural language processing [13], machine vision [14,15], and time series [16]. These successes can be attributed to the ability of self-attention in transformers to obtain long-range individual relationships. Furthermore, following the observation in [17,18], it is evident that time series data undergo distribution shifts. Addressing distribution shifts while performing time series forecasting has led to significantly improved results [17,18]. However, attempting to normalize time series data during anomaly detection could further degrade the performance of the model owing to the sparsity of anomalies in actual data. Also, normalizing time series data, like in [17], could nullify the anomalies as anomalous datapoints will be closer in value to the normal datapoints. We normalize the used time series dataset for anomaly detection such that anomalies are highlighted as compared to normal datapoints. In anomaly transformers [12], the idea of using prior associations and series-associations seems to be highly effective. Here, series associations are calculated using the self-attention of transformers, and prior associations are calculated using learnable Gaussian kernels to calculate priors with respect to the relative temporal distance. To that end, reversibly normalized data should be used for determining series associations, and regular datapoints should be passed to determine prior associations. In this way, when a minimax-strategy-based association discrepancy is used for anomaly detection, the anomalies are highlighted more as compared to normal datapoints. The contributions of the paper can be summarized as follows:

- the suggestion of the reversible instance normalized anomaly transformer to highlight anomalies better than normal datapoints;
- the achievement of comparable or better results in four actual datasets.

## 2. Related Works

Anomalies in time series data can occur in various ways and can broadly be categorized into temporal, intermetric, or a combination of temporal–intermetric anomalies. Temporal anomalies [19] can be global, where singular or multiple points in a series have values significantly different from the rest. Contextual anomalies are variations relative to neighboring data points. Anomalies in one context might be normal in another. Seasonal anomalies deviate from the regular seasonality of a series. Trend anomalies cause a persistent shift in the data’s mean, leading to a change in the time series trend without affecting its cyclical and seasonal patterns. Shapelets pertain to subsequences in data for which cycles or shapes deviate from the usual pattern, influenced by external factors. In analyzing time series data, several algorithms have been proposed for anomaly detection. Based on intrinsic model characteristics, these anomaly detection algorithms can be systematically classified into five distinct categories.

### 2.1. Stochastic Models

Although modern machine-learning-based methods are increasingly popular for this task, there are several traditional techniques and categories that have been used over the years. These models operate on the assumption that data follow a specific statistical pattern or distribution. Anomalies are identified when observed data points deviate significantly from this expected pattern. Examples include autoregressive integrated moving average (ARIMA) [20], the exponential smoothing state space model (ETS) [21], and the seasonal decomposition of time series (STL) [22].

### 2.2. Distance-Based Models

The core idea of these models is that anomalies are data points that are far away from other points. Examples include the k-nearest neighbor (k-NN) algorithm [23], where a point is considered an anomaly if its distance from its k^th^ nearest neighbor exceeds some threshold. Density-based methods, like (DBSCAN) [24], can also be considered in this category, where sparse regions with a low density of data points can be indicative of anomalies.

### 2.3. Information-Theoretic Models

These models are based on concepts from information theory, such as entropy. The idea is to measure the randomness or unpredictability in the data [25]. High or low entropy regions, depending on the context, can be indicative of anomalies. A sudden spike in entropy in time series data might indicate an anomaly.

### 2.4. Machine Learning and Deep Learning Models

These models are trained on historical time series data to learn data patterns. Anomalies are detected when new data points significantly differ from the model’s prediction. We can further divide machine learning and deep learning models into two categories, namely, forecasting-based models and reconstruction-based models.

### 2.5. Forecasting-Based Models

Forecasting-based models learn the usual patterns from past data, predict future patterns, and then label anomalies if real future data are too different from their predictions. Recurrent neural networks (RNNs) are the commonly used approach as they are designed to handle sequences of data, making them naturally suited for time series. RNNs are trained on a sequence of data points to learn the pattern. When predicting future data points, if the actual data deviate significantly from their predictions, the data are labeled as anomalous. Long short-term memory (LSTM) is an advanced type of RNN that is designed to remember patterns over long sequences and avoid long-term dependency issues found in traditional RNNs. LSTMs are particularly good at capturing long-term patterns in time series data. If the LSTM’s prediction for a future data point does not match the actual observed data, it is an indication of an anomaly. Owing to their long memory, they can be particularly useful for spotting anomalies that are based on long-term patterns [26,27]. Convolutional neural networks (CNNs) are primarily designed for image processing to identify spatial hierarchies in data. However, they can be adapted for time series data by treating segments of time series as local patterns. A CNN can slide over a time series and learn local patterns [28]. After training, if a new pattern appears that does not match any learned pattern, the CNN can label this as an anomaly. It is effective for capturing local anomalies in a dataset. Transformer-based models [29] use attention mechanisms to weigh the importance of different data points in a sequence. Introduced for natural language-processing tasks, their adaptability has extended their usage for time series forecasting. Transformers can give attention to significant patterns in a time series dataset. When trained, if the model encounters a data point or sequence that significantly deviates from the patterns it gave attention to, the model can label that as an anomaly. The capacity to handle long sequences with varied attention spans makes transformers robust for complex anomaly detection scenarios. Graph neural networks (GNNs) are designed for graph-structured data. Graphs consist of nodes and edges, and GNNs process these data by propagating and aggregating information from neighboring nodes to enhance the feature representation of each node or edge. Time series data are transformed into a graph format, especially when there is a relationship or correlation between different time series. For instance, in multivariate time series, where different series influence each other, or in scenarios where temporal patterns form a network of relationships [30], GNNs learn the underlying structure and relationships in the data. When a deviation from the learned graph structure or relationship pattern occurs, it is an indication of an anomaly.

### 2.6. Reconstruction-Based Models

This type of model aims to learn a compressed representation of the data and then reconstruct it. Anomalies are often identified based on how well the model can reconstruct a particular data point or sequence. Autoencoder-based models [31] aim to copy their inputs to their outputs and consist of an encoder, which compresses the input into a latent-space representation, and a decoder, which reconstructs the input data from this representation. For anomaly detection, this model trains the autoencoder on normal data so that it learns to reconstruct the input data well. When an anomalous data point is passed through, the reconstruction error (difference between the original data point and its reconstruction) tends to be high, signaling an anomaly. Variational autoencoder (VAE)-based models [32] are a type of autoencoder with added constraints on the encoded representations and are designed to generate new data points and, hence, are often used in generative tasks. For anomaly detection, like standard autoencoders, VAEs are trained on normal data to learn the data structure. Anomalies are data points that are difficult for the VAE to reconstruct, leading to high reconstruction errors. Additionally, the latent space of a VAE (where data are compressed) follows a specific distribution, and deviations from this can also signal anomalies. Generative adversarial network (GAN)-based models [33] consist of two networks: a generator that produces data and a discriminator that evaluates them. The generator tries to produce data that the discriminator cannot distinguish from real data. GANs can be trained on normal data, where the generator learns to produce normal data samples. When a real data point is fed to the discriminator and is deemed as “fake” (or different from the learned distribution), it can be an indication of an anomaly [34].

## 3. Proposed Method

We propose an anomaly detection method that combines a transformer architecture with an autoencoder structure. Transformer-based models are originally designed for natural language-processing tasks [11]. These models use an attention mechanism to weigh the importance of different data points in a sequence, enabling the models to capture long-range dependencies in data. Transformers can be trained in a reconstruction manner similar to autoencoders and can learn to predict or reconstruct a segment of a time series based on its context. A high reconstruction error indicates an anomaly. Given the transformer’s ability to handle long sequences and varied attention spans, it can capture both local and global anomalies in data. In the majority of existing time series anomaly detection methods, there is a prevalent emphasis on understanding predominant temporal patterns. However, these traditional approaches prioritize either pointwise representations focusing on individual data points or pairwise associations examining relationships between pairs. Thus, these models often hesitate in comprehensively capturing the adjacent concentration inductive bias of each time point in time series data. This inductive bias suggests that for each time point in a time series, its immediate neighbors are more relevant or influential for its representation than distant points. Furthermore, these models can be susceptible to distribution shifts in the data, meaning that the models might struggle when the underlying statistical properties of the time series change over time.

To address the challenges faced by traditional time series anomaly detection methods, a two-fold solution is proposed. First, the learnable Gaussian kernel is introduced to effectively handle the adjacent concentration inductive bias, ensuring that each data point in the series adequately emphasizes its immediate neighbors. Second, the integration of reversible instance normalization (RevIN) is suggested, incorporating both normalization and denormalization with a learnable affine transformation. This approach provides a robust mechanism to counteract distribution shifts, ensuring consistent model performance even as the underlying statistical properties of the data evolve.

### 3.1. Anomaly Transformer

The anomaly transformer is an adaptation of the transformer architecture designed for unsupervised time series anomaly detection. In anomaly transformers, the temporal association between data from each time point is obtained using a self-attention map and is termed as ‘series association’. The series association is more significant for non-anomalous time points and less so for anomalous time points. As anomalous time points are less frequent, their associations with the adjacent time points are much higher, where these disruptions are more likely to appear. This is termed as ‘prior association’. Based on the series association and prior association, a new criterion called the ‘association discrepancy’ is introduced for anomaly classification. The self-attention is modified to separately obtain the prior association and series association for each time point. Although series associations are obtained using the conventional self-attention, prior associations are obtained using learnable Gaussian kernels. A minimax approach is implemented to enhance the differentiation between normal and abnormal patterns in the association discrepancy.

### 3.2. Reversible Instance Normalization

Time-series forecasting models frequently encounter challenges related to distribution shifts, where statistical properties in training and test data evolve over time, leading to performance issues. Although removing non-stationary information from input sequences can mitigate these discrepancies, it may compromise the model’s ability to capture the original data distribution. To address this issue, reversible instance normalization (RevIN) was introduced, a method that normalizes input sequences and then denormalizes the model’s output sequences using normalization statistics [17]. This approach maintains the performance while effectively handling distribution shifts in time-series forecasting.

Suppose we have a set of input and output time series data, $X={\left\{x^{\left(i\right)}\right\}}_{i=1}^{N}$ and $Y={\left\{y^{\left(i\right)}\right\}}_{i=1}^{N}$, respectively, where *N* is the number of sequences, *K* is the number of variables, *T_x_* is the length of the input, and *T_y_* is the length of the output. Then, given the mean and standard deviation of each instance, $x_{k}^{\left(i\right)}\in R^{T_{x}}$, the data are normalized as follows:

(1) $${\hat{x}}_{kt}^{\left(i\right)}=\gamma_{k}\left(\frac{x_{kt}^{\left(i\right)}-{µ}_{t}\left[x_{kt}^{\left(i\right)}\right]}{\sqrt{Var\left[x_{kt}^{\left(i\right)}\right]+\varepsilon }}\right)+\beta_{k}$$

where ${µ}_{t}\left[x_{kt}^{\left(i\right)}\right]$ and $Var\left[x_{kt}^{\left(i\right)}\right]$ are the mean and standard deviation (Var), respectively, and γ$,\;\beta \;\in R^{K}$ are learnable affine parameters. The mean and standard deviation are given as follows:

(2) $${µ}_{t}\left[x_{kt}^{\left(i\right)}\right]=\frac{1}{T_{x}}\sum_{j=1}^{T_{x}}x_{kj}^{\left(i\right)}\;\mathrm{and}\;Var\left[x_{kt}^{\left(i\right)}\right]=\frac{1}{T_{x}}\sum_{j=1}^{T_{x}}{{(x}_{kj}^{\left(i\right)}-{µ}_{t}\left[x_{kt}^{\left(i\right)}\right])}^{2}$$

Similarly, the forecasting-model output is denormalized as follows:

(3) $${\hat{y}}_{kt}^{\left(i\right)}=\sqrt{Var\left[x_{kt}^{\left(i\right)}\right]+\varepsilon }\left(\frac{{\tilde{y}}_{kt}^{\left(i\right)}-\beta_{k}}{\gamma_{k}}\right)+{µ}_{t}\left[x_{kt}^{\left(i\right)}\right]$$

In this work, we intentionally used the concept of normalization to further emphasize the differences between the anomalous and non-anomalous datapoints by normalizing the data. Because anomalies are rare, it is difficult for them to build series associations, and their associations with their neighboring datapoints are stronger. When input data are normalized, anomalies in the data are less significant. Considering this, we propose to find series associations using normalized data and prior associations using the original (non-normalized) data. We hypothesize that this way, stronger prior associations can be observed, which will help us to obtain better association discrepancies. In our architecture, we do not use learnable parameters, β, as it has previously been determined that the difference between using them and not using them is negligible [18].

### 3.3. Reversible Instance Normalized Anomaly Transformer (RINAT)

**Figure 1:**
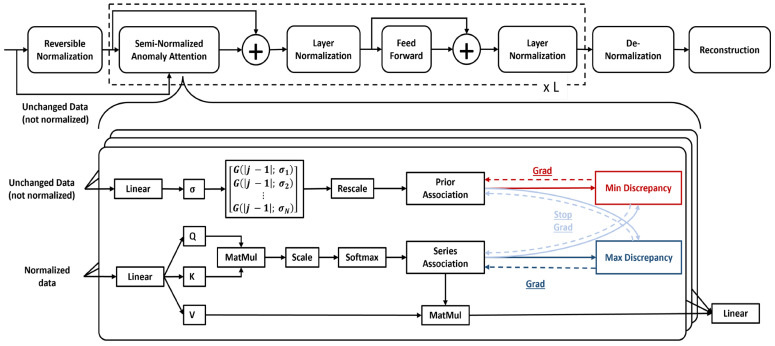
Reversible instance normalized anomaly transformer (RINAT).

By focusing on the constraints of transformers and the achievement of the anomaly transformer in unsupervised anomaly detection, we enhanced the anomaly transformer to the reversible instance normalized anomaly transformer. We adopted the anomaly transformer [12] as it addresses the challenge of the adjacent inductive by introducing the prior association and series association of each time point. We also leveraged the concept of the reversible normalization and rethought the anomaly transformer for the same application. This architecture estimates the anomaly score based on the association discrepancy and reconstruction error. The association discrepancy considers the prior association and series association of each time point. The prior association employs the learnable Gaussian kernel to present the adjacent concentration inductive bias of each time point. The series association corresponds to the self-attention weights learned from raw series. We renovated the anomaly transformer by adding the reversible instance learnable normalization to input time series data because anomalies are rare and normalization might reduce the impact of anomalies. Thus, normalization was only applied to the series association part, as shown in Figure 1. This partial application of the reversible instance normalization brings to light the variations between the series associations and the prior associations while determining the association discrepancies. As in the anomaly transformer, we utilized an encoder-only design, with stacks of specially designed attention blocks and feedforward layers. These stacks are repeated multiple times. However, the attention block is different from the anomaly attention block.

Given the time series data, $X\in R^{T}$, with T time steps, and each time-step value, $x_{i}$, in the sequence, we perform embedding on the given time series data. For the input layer, we take layer l=0.

(4) $$X_{Out}^{l=0}=emb(X)$$

For $X_{Out}^{l=0}\in R^{T\times D}$, D represents the embedding dimension, effectively capturing both the time series length and the embedded feature dimensions. The proposed transformer architecture for anomaly detection integrates the power of the traditional transformer with additional steps. These steps include the reversible normalization, semi-stationary anomaly attention, as well as strategic placements of the layer normalization and denormalization. A salient feature of this architecture is the semi-stationary anomaly attention, which intakes two distinct inputs. The first one is the normalized data from the reversible normalization stage and second one is the raw embedded data directly from the embedding phase. The reversible normalization stage normalizes the given data by subtracting their mean, μ, and dividing by their standard deviation, σ.

(5) $$X_{norm}^{l=0}=\frac{X_{Out}^{l=0}-\mu }{\sigma }$$

Given two distinct inputs to the semi-stationary anomaly attention, this stage estimates the anomaly discrepancy using the two-branch structure. One branch estimates the prior association to address the challenge of the adjacent inductive. The relationship between two temporal points, i and j, with respect to the relative temporal distance within the series is quantified using the Gaussian kernel, represented by the following equation:

(6) $$P^{l}=rescale({\left[\frac{1}{\sqrt{2\pi }\sigma_{i}}exp(-\frac{{\left|j-i\right|}^{2}}{2\sigma_{i}^{2}})\right]}_{i,j\in \{1,2\ldots N\}})$$

Benefiting from the unimodal property of the Gaussian kernel, essentially, this design can pay more attention to the adjacent time points. The learnable scale parameter, σ, for the Gaussian kernel makes prior associations adapt to various time series patterns, such as different lengths of anomaly segments.

Next, a branch of the normalized anomaly attention estimates the series association. The series association corresponds to the self-attention weights learned from raw series. Given the embedded data, $X_{norm}^{l=0}$, self-attention weights are computed using a scaled dot-product between query $Q_{l}$, and key $K_{l}$, followed by a SoftMax operation. We compute $Q_{l}$, $K_{l}$, and $V_{l}$ as follows:

(7) $$Q_{l}=X_{norm}^{l=0}W_{Q}^{l}\;K_{l}=X_{norm}^{l=0}W_{K}^{l}\;V_{l}=X_{norm}^{l=0}W_{V}^{l}$$

where $W_{Q}^{l}\in R^{d_{v}\times d_{q}}$, $W_{K}^{l}\in R^{d_{v}\times d_{k}}$, and $W_{V}^{l}\in R^{d_{v}\times d_{v}}$ are weights for layer l. Then, the series association coefficient, $S^{l}$, is derived as follows:

(8) $$S^{l}=SoftMax(\frac{Q^{l}K^{lT}}{\sqrt{d_{k}}})$$

(9) $$X_{Out}^{l}=S^{l\;}\times V^{l}$$

The series association coefficient and prior association coefficient both represent the probability distribution. The disparity between the prior and series associations is measured using the Kullback–Leibler (KL) divergence.

(10) $$AssDisp(P,S;X)={(\frac{1}{L}[\sum_{l=1}^{L}(KL(P_{i}^{l}||S_{i}^{l}+KL(S_{i}^{l}||P_{i}^{l}))])}_{i=1,2,3\ldots N}$$

After the attention mechanism, the output is normalized using the layer normalization. This step improves the model convergence and ensures stable activations. The normalized output is then directed to a feedforward neural network, which further extracts high-level features and representations from the data. Once processed, the output undergoes another layer normalization step to maintain a stabilized activation range. To preserve the original time series scale and pattern, a denormalization step is employed, reversing the effects of the initial normalization and ensuring the final output remains intricately tied to the original series dynamics.

(11) $$X_{Rev}=\frac{X_{norm}^{rec}-\beta }{\gamma }$$

In the proposed architecture, the process for learning or training to achieve the desired performance is guided using two loss functions simultaneously. This dual-loss approach helps the network to learn and adapt based on two different objectives or criteria. The primary component is the reconstruction loss, measuring the disparity between the original series and the decoded output, essentially guiding the series association to recognize the most pivotal associations. Complementing this is the association discrepancy loss, which highlights the differences between typical patterns and unusual patterns in time series data. The loss function for input series is as follows:

(12) $${\mathrm{Loss}}_{\mathrm{Final}\;}(X,P,S,\lambda ;X_{Rev})=∥X-X_{Rev}∥-\lambda ∥AssDisp(P,S;X)∥$$

The value of λ determines the influence of the association discrepancy within the broader context of the loss function. Additionally, we implemented the minimax strategy to make the association discrepancy more distinguishable. This approach is employed between the series association and prior association in two phases. In the minimize phase, the model adjusts the prior association, $P^{l}$, to reflect the series association, $S^{l}$. The prior association serves as an initial model or understanding, which is then refined or updated based on the actual patterns observed in the series association. This enables the prior association to become more adaptable to a variety of temporal patterns found in the data. Conversely, in the maximize phase, the objective is to increase the association discrepancy, pushing the series association to focus more on non-adjacent data points. The model pays extra attention to data points that are separated by significant time intervals. A score, AS(X), is assigned for each data point in the series to quantify the deviation of the data point from the norm.

(13) $$AS(X)=SoftMax(-AssDisp(P,S;X_{\mathrm{norm}\;}))⊙∥X-X_{Rev}∥$$

This gives the pointwise anomaly criteria based on the association discrepancy.

## 4. Experiments

We extensively evaluated the proposed RINAT with different publicly available datasets in three practical applications.

### 4.1. Datasets

We used the following four datasets in our experiments: (1) the server machine dataset (SMD) [35], which is a dataset collected from a large internet company and consists of five-week-long data with 38 dimensions; (2) pooled server metrics (PSMs) [36], which are a collection of internally collected data from multiple application server nodes at eBay and have 26 dimensions; and the (3) Mars Science Laboratory (MSL) rover [37] and (4) Soil Moisture Active Passive (SMAP) [37] satellite datasets, which are public datasets made available by NASA, contain telemetry anomaly data derived from Incident Surprise Anomaly (ISA) reports of spacecraft monitoring systems, and have 55 and 25 dimensions, respectively.

### 4.2. Implementation Details

The overall experiments were performed in a system with a single Nvidia Geforce RTX 3090, and the implemented code was written in the Pytorch framework of version 1.13. The overall setup was implemented in a fashion similar to that in the work of the anomaly transformer [12]. A non-overlapping sliding window was used to obtain a set of sub-series, just like in [38]. For all the datasets, the sliding window was set to a fixed size of 100. Time points were labeled as anomalies if their anomaly scores were higher than a certain threshold, δ. The threshold, δ, was determined such that a proportion, r, of the data in the validation dataset would be labeled as anomalies. For the SMD dataset, we set r = 0.5% and 1% for the rest. For anomaly detection, if a single time point in a certain segment of an anomalous time series was detected, it was considered that the whole anomalous segment was detected. This adjustment strategy has previously been widely adopted [35,38,39] and, similar to the adjustment strategy for the anomaly transformer [12], contains three layers. We set the number of channels in the hidden-state model at 512 and the number of heads, h, at 8. The hyperparameter, λ, (Equation (4)) was set at 3 for all the datasets to tradeoff two parts of the loss function. We used the ADAM optimizer [40] at an initial learning rate of 10^−4^. The training process was stopped early, within 10 epochs, with a batch size of 32.

### 4.3. Baselines

We compared our model with 16 other baseline models, namely, InterFusion [41], BeatGAN [42], OmniAnomaly [35], LSTM-VAE [32], DAGMM [43], MPPCACD [44], LOF [45], ITAD [46], THOC [38], Deep-SVDD [47], CL-MPPCCA [48], LSTM [37], VAR [49], OC-SVM [50], IsolationForest [51], and the anomaly transformer [12].

### 4.4. Results

**Figure 2:**
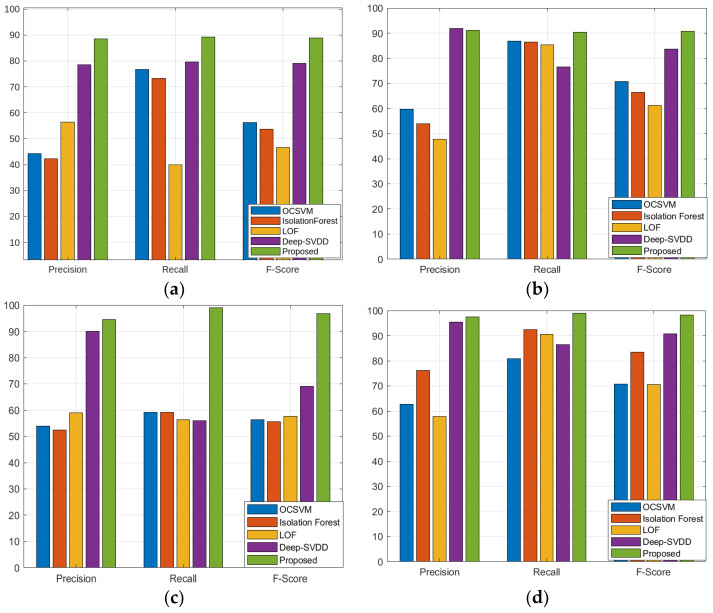
Comparison of the proposed model with four different models in group1 classifiers using four different datasets: (**a**) SMD; (**b**) MSL; (**c**) SMAP; (**d**) PSM.

**Figure 3:**
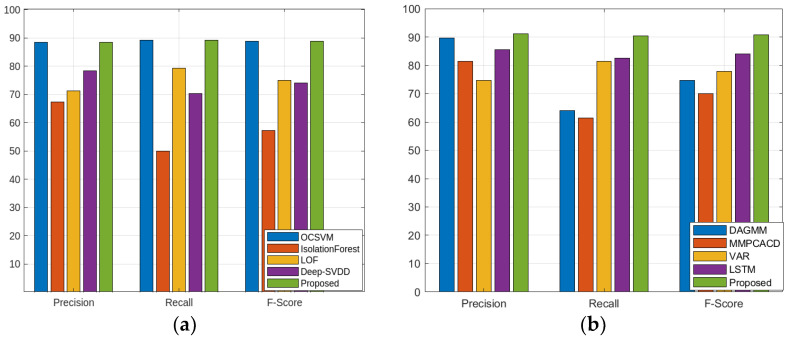

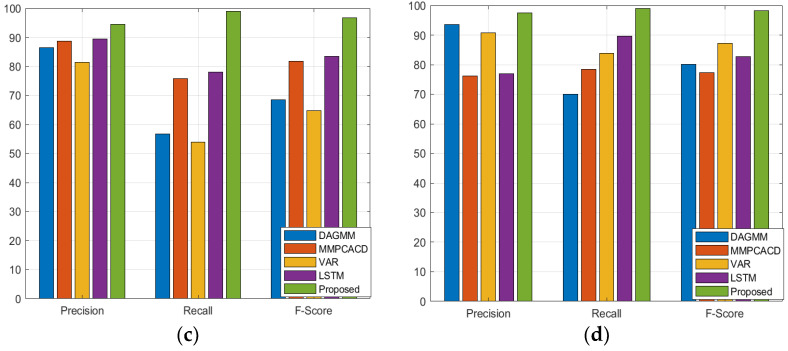
Comparison of the proposed model with four different models in group2 classifiers using four different datasets: (**a**) SMD; (**b**) MSL; (**c**) SMAP; (**d**) PSM.

**Figure 4:**
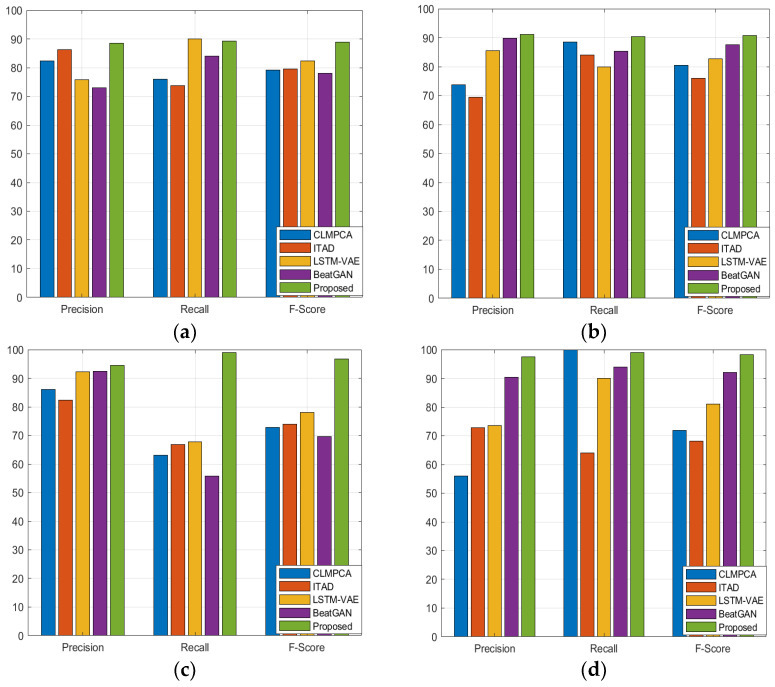
Comparison of the proposed model with four different models in group3 classifiers using four different datasets: (**a**) SMD; (**b**) MSL; (**c**) SMAP; (**d**) PSM.

**Figure 5:**
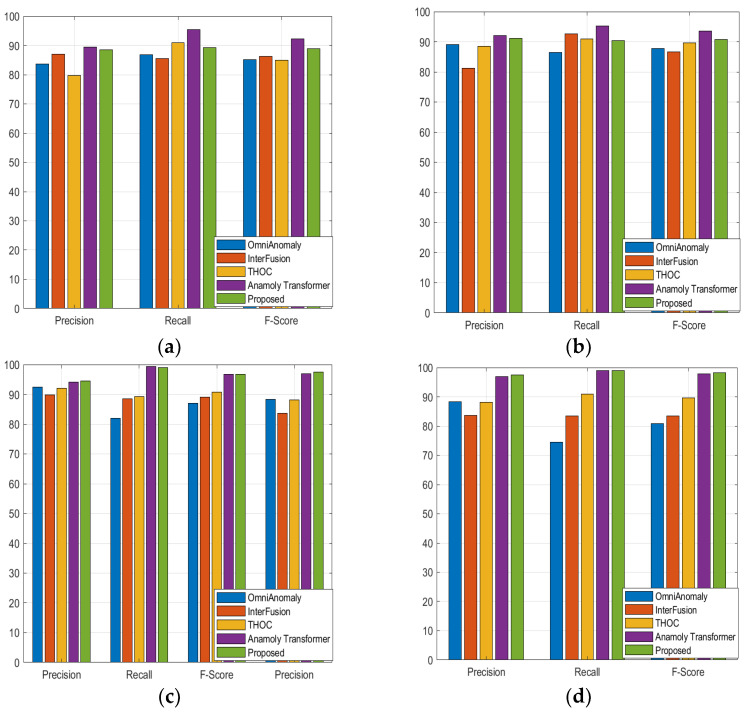
Comparison of proposed model with four different models in group4 classifiers using four different datasets: (**a**) SMD; (**b**) MSL; (**c**) SMAP; (**d**) PSM.

Table 1 shows the quantitative comparison of the precision, recall, and F1 scores for the 16 other baseline models and the suggested model. We can see that although the performance of the suggested model is comparable to that of the anomaly transformer in the SMD, MSL, and SMAP datasets, it is better than that of the state-of-the-art anomaly transformer in the PSM dataset. Figure 2, Figure 3, Figure 4 and Figure 5 show the comparisons of the precision, recall, and F1 scores, respectively. The proposed model outperforms almost all the existing algorithms except for the anomaly transformer.

**Figure 6:**
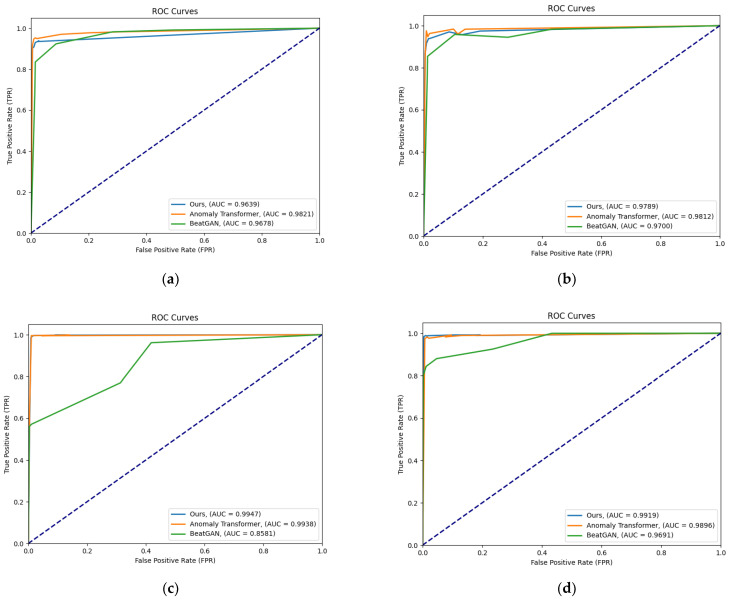
ROC curves (horizontal axis: false-positive rate; vertical axis: true-positive rate) for four different datasets: (**a**) SMD; (**b**) MSL; (**c**) SMAP; (**d**) PSM. A higher AUC value (area under the ROC curve) indicates a better performance. The predefined threshold proportion, r, is in {0.5%, 1.0%, 1.5%, 2.0%, 10%, 20%, and 30%}.

With the MSL data, the proposed model shows a slightly lower performance compared to that of the anomaly transformer, especially in terms of the precision and F1 scores. Although the performance of the proposed model is impressive and slightly better than that of the anomaly transformer, with an F1 score of 98.28, we can see that the performance of the proposed model is very comparable to that of the anomaly transformer. The F1 scores are almost the same, indicating a similar overall performance in this dataset. The proposed model seems to show a drop in performance, especially in terms of the recall and F1 scores, compared to those of the anomaly transformer. The anomaly transformer tends to perform better than the proposed model in the MSL and SMD datasets in terms of the F1 score, while the proposed model has a slight edge over the anomaly transformer in the PSM dataset. Both models perform similarly in the SMAP dataset. The proposed model consistently shows higher precision than the anomaly transformer in all the datasets, but it tends to have a lower recall score than the anomaly transformer in the MSL and SMD datasets. Figure 6 shows the ROC curves for the suggested model architecture alongside the ROC curves of the anomaly transformer and BeatGAN architectures. The AUC values of the suggested model architecture in the SMAP and PSM datasets seem to be better than those of even the anomaly transformer architecture. Additionally, for the MSL and SMD datasets, even though the proposed model architecture does not outshine that of the anomaly transformer, the AUC values are comparable.

## 5. Conclusions

In conclusion, our paper introduced the reversible instance normalized anomaly transformer, building upon the fundamental principles of the anomaly transformer. Through a comprehensive evaluation of well-established benchmarks, including those of the anomaly transformer and 16 other baseline models across multiple datasets, we have gained valuable insights. Although our model demonstrates commendable performance, it is crucial to recognize that the model’s strengths and limitations are context-dependent, varying across datasets.

This variability in performance underscores the importance for considering the specific characteristics and complexities of each dataset. Notably, the proposed model exhibits a decline in the recall score in comparison to that of the anomaly transformer in the MSL and SMD datasets, which suggests a potentially higher rate of false negatives for these datasets. However, when considering the F1 score, which provides a balanced view by combining precision and recall scores, our proposed model holds a slight advantage over the anomaly transformer in the PSM dataset. On the other hand, the anomaly transformer outperforms the proposed model in the MSL and SMD datasets. Interestingly, the performance remains comparable for both models in the SMAP dataset.

Although our research contributes a valuable approach to anomaly detection, its effectiveness is subject to the unique characteristics of each dataset. These findings underscore the need for further research to adapt and fine-tune anomaly detection models for specific domains, thereby enhancing their applicability in a diverse range of actual scenarios. Future work should focus on addressing the limitations of our model, particularly in datasets where recall is a critical metric, and optimizing it for broader applicability. Additionally, a deeper theoretical exploration of the model’s validity and further refinement may open doors for improving its performance in challenging datasets, like MSL and SMD.

## Figures and Tables

**Table 1:** Quantitative results for the suggested model and 16 other models in four actual datasets. The metrics used for comparison are precision (P), recall (R), and F1 scores. Higher values represent better performance in each of these metrics. The results of anomaly transformer was replicated using their provided code while for the rest of models the results were copied from the anomaly transformer paper [12].

Dataset	SMD			MSL			SMAP			PSM		
Metric	P	R	F1	P	R	F1	P	R	F1	P	R	F1
OCSVM	44.34	76.72	56.19	59.78	86.87	70.82	53.85	59.07	56.34	62.75	80.89	70.67
IsolationForest	42.31	73.29	53.64	53.94	86.54	66.45	52.39	59.07	55.53	76.09	92.45	83.48
LOF	56.34	39.86	46.68	47.72	85.25	61.18	58.93	56.33	57.60	57.89	90.49	70.61
Deep-SVDD	78.54	79.67	79.10	91.92	76.63	83.58	89.93	56.02	69.04	95.41	86.49	90.73
DAGMM	67.30	49.89	57.30	89.60	63.93	74.62	86.45	56.73	68.51	93.49	70.03	80.08
MMPCACD	71.20	79.28	75.02	81.42	61.31	69.95	88.61	75.84	81.73	76.26	78.35	77.29
VAR	78.35	70.26	74.08	74.68	81.42	77.9	81.38	53.88	64.83	90.71	83.82	87.13
LSTM	78.55	85.28	81.78	85.45	82.50	83.95	89.41	78.13	83.39	76.93	89.64	82.80
CL-MPPCA	82.36	76.07	79.09	73.71	88.54	80.44	86.13	63.16	72.88	56.02	99.93	71.80
ITAD	86.22	73.71	79.48	69.44	84.09	76.07	82.42	66.89	73.85	72.80	64.02	68.13
LSTM-VAE	75.76	90.08	82.30	85.49	79.94	82.62	92.20	67.75	78.10	73.62	89.92	80.96
BeatGAN	72.90	84.09	78.10	89.75	85.42	87.53	92.38	55.85	69.61	90.30	93.84	92.04
OmniAnomaly	83.68	86.82	85.22	83.02	86.37	87.67	92.49	81.99	86.92	88.39	74.46	80.83
InterFusion	87.02	85.43	86.22	81.28	92.70	86.62	89.77	88.52	89.14	83.61	83.45	83.52
THOC	79.76	90.95	84.99	88.45	90.97	89.69	92.06	89.34	90.68	88.14	90.99	89.54
Anomaly Transformer	89.40	95.45	92.33	92.09	95.15	93.59	94.13	99.40	96.69	96.91	98.90	97.89
Our Model	88.56	89.29	88.92	91.06	90.29	90.68	94.40	99.04	96.67	97.52	99.06	98.28

## Data Availability

The code and dataset shall be made available upon request via email to the corresponding author.
